# Tobacco Product Use Among Middle and High School Students — United States, 2011–2017

**DOI:** 10.15585/mmwr.mm6722a3

**Published:** 2018-06-08

**Authors:** Teresa W. Wang, Andrea Gentzke, Saida Sharapova, Karen A. Cullen, Bridget K. Ambrose, Ahmed Jamal

**Affiliations:** ^1^Office on Smoking and Health, National Center for Chronic Disease Prevention and Health Promotion, CDC; ^2^Center for Tobacco Products, Food and Drug Administration, Silver Spring, Maryland.

Tobacco use is the leading cause of preventable disease and death in the United States, and nearly all tobacco use begins during youth and young adulthood (*1*,*2*). CDC and the Food and Drug Administration (FDA) analyzed data from the 2011–2017 National Youth Tobacco Surveys (NYTS)* to determine patterns of current (past 30-day) use of seven tobacco product types among U.S. middle school (grades 6–8) and high school (grades 9–12) students and estimate use nationwide. Among high school students, current use of any tobacco product decreased from 24.2% (estimated 3.69 million users) in 2011 to 19.6% (2.95 million) in 2017. Among middle school students, current use of any tobacco product decreased from 7.5% (0.87 million) in 2011 to 5.6% (0.67 million) in 2017. In 2017, electronic cigarettes (e-cigarettes) were the most commonly used tobacco product among high (11.7%; 1.73 million) and middle (3.3%; 0.39 million) school students. During 2016–2017, decreases in current use of hookah and pipe tobacco occurred among high school students, while decreases in current use of any tobacco product, e-cigarettes, and hookah occurred among middle school students. Current use of any combustible tobacco product, ≥2 tobacco products, cigarettes, cigars, smokeless tobacco, and bidis did not change among middle or high school students during 2016–2017. Comprehensive and sustained strategies can help prevent and reduce the use of all forms of tobacco products among U.S. youths (*1*,*2*).

NYTS is a cross-sectional, voluntary, school-based, self-administered, pencil-and-paper questionnaire survey of U.S. middle and high school students. A three-stage cluster sampling procedure is used to generate a nationally representative sample of U.S. students attending public and private schools in grades 6–12. Briefly, primary sampling units are selected at the first stage, schools are selected at the second stage, and students are selected from intact classrooms at each grade level at the third stage. This report used data from seven NYTS waves (2011–2017). Sample sizes and response rates were 18,766, 72.7% (2011); 24,658, 73.6% (2012); 18,406, 67.8% (2013); 22,007, 73.3% (2014); 17,711, 63.4% (2015); 20,675, 71.6% (2016); and 17,872, 68.1% (2017).

Participants were asked about current (past 30-day) use of cigarettes, cigars, smokeless tobacco,^†^ e-cigarettes,^§^ hookah,^¶^ pipe tobacco,** and bidis (small imported cigarettes wrapped in a leaf). Current use for each product was defined as use on ≥1 day during the past 30 days. “Any tobacco product use” was defined as use of one or more tobacco products in the past 30 days, and “≥2 tobacco product use” was defined as use of two or more tobacco products in the past 30 days. “Any combustible tobacco product use” was defined as use of cigarettes, cigars, hookah, pipe tobacco, and/or bidis in the past 30 days.

Data were weighted to account for the complex survey design and adjusted for nonresponse. National prevalence estimates with 95% confidence intervals and population estimates rounded down to the nearest 10,000 were computed. Current use estimates for 2017 were determined for any tobacco product, ≥2 tobacco products, any combustible tobacco product, and each tobacco product individually, overall and by selected demographics for each school level (high and middle). The presence of linear and quadratic trends during 2011–2017 were assessed, adjusting for race/ethnicity, sex, and grade.^††^ T-tests were performed to examine differences between 2016 and 2017. For all analyses, p-values <0.05 were considered statistically significant.

In 2017, 19.6% of high school students (estimated 2.95 million users) reported current use of any tobacco product, including 9.2% (1.38 million; 46.8% of current tobacco product users) who currently used ≥2 tobacco products, and 12.9% (1.94 million; 65.8% of current tobacco product users) who currently used any combustible tobacco product (Table). E-cigarettes were the most commonly used tobacco product among high school students (11.7%), followed by cigars (7.7%), cigarettes (7.6%), smokeless tobacco (5.5%), hookah (3.3%), pipe tobacco (0.8%), and bidis (0.7%). Smokeless tobacco use was higher among males than among females. E-cigarettes were the most commonly used tobacco product among non-Hispanic white (white) (14.2%) and Hispanic (10.1%) high school students, whereas cigars were the most commonly used tobacco product among non-Hispanic black (black) high school students (7.8%).

**TABLE:** Estimated prevalence of tobacco use among high school and middle school students in the past 30 days, by product,* school level, sex, and race/ethnicity**^†^** — National Youth Tobacco Survey, United States, 2017

Tobacco product	Sex		Race/Ethnicity				Total	
	Female	Male	White^†^	Black^†^	Hispanic	Other^†^		
	% (95% CI)	% (95% CI)	% (95% CI)	% (95% CI)	% (95% CI)	% (95% CI)	% (95% CI)	Estimated no. users^§^
**High school students**								
E-cigarettes	9.9 (8.0–12.1)	13.3 (11.1–15.9)	14.2 (12.2–16.5)	4.9 (3.5–6.8)	10.1 (7.0–14.4)	5.5 (3.1–9.5)	**11.7 (9.7–13.9)**	**1,730,000**
Cigarettes	7.5 (6.1–9.2)	7.6 (6.4–9.0)	9.5 (8.0–11.3)	2.8 (1.7–4.4)	6.2 (4.6–8.3)	3.8 (2.2–6.2)	**7.6 (6.5–8.9)**	**1,120,000**
Cigars	6.3 (5.0–7.8)	9.0 (7.6–10.7)	8.4 (6.9–10.0)	7.8 (5.8–10.4)	6.7 (5.1–8.6)	4.1 (2.6–6.3)	**7.7 (6.5–9.0)**	**1,130,000**
Smokeless tobacco	3.0 (2.3–4.0)	7.7 (5.9–10.0)	7.2 (5.6–9.4)	1.8 (1.2–2.8)	3.7 (2.6–5.3)	—^¶^	**5.5 (4.2–7.0)**	**810,000**
Hookah	3.2 (2.5–4.1)	3.3 (2.5–4.3)	2.8 (2.1–3.7)	3.1 (2.3–4.3)	4.6 (3.4–6.3)	3.3 (2.1–5.1)	**3.3 (2.7–4.0)**	**480,000**
Pipe tobacco	0.5 (0.4–0.8)	1.0 (0.8–1.4)	0.7 (0.5–1.1)	—	1.3 (0.8–2.0)	—	**0.8 (0.6–1.0)**	**120,000**
Bidis	0.6 (0.4–0.9)	0.7 (0.4–1.1)	0.5 (0.3–0.8)	—	1.1 (0.7–1.7)	—	**0.7 (0.5–1.0)**	**100,000**
Any tobacco product**	17.5 (15.2–20.1)	21.5 (18.7–24.6)	22.7 (20.3–25.4)	14.2 (11.6–17.3)	16.7 (12.9–21.4)	10.7 (7.0–16.2)	**19.6 (17.2–22.3)**	**2,950,000**
≥2 tobacco products^††^	7.6 (6.2–9.4)	10.7 (9.0–12.6)	11.3 (9.6–13.2)	4.4 (3.1–6.2)	8.2 (5.9–11.3)	4.0 (2.6–6.2)	**9.2 (7.8–10.9)**	**1,380,000**
Any combustible tobacco product^§§^	12.2 (10.4–14.2)	13.5 (11.6–15.6)	14.4 (12.4–16.5)	10.9 (8.7–13.6)	11.8 (9.2–15.1)	6.8 (4.4–10.3)	**12.9 (11.2–14.8)**	**1,940,000**
**Middle school students**								
E-cigarettes	2.9 (2.3–3.7)	3.7 (3.0–4.5)	3.4 (2.6–4.5)	2.2 (1.3–3.6)	4.0 (2.9–5.5)	—	**3.3 (2.8–3.9)**	**390,000**
Cigarettes	2.2 (1.7–2.9)	2.0 (1.5–2.8)	1.7 (1.3–2.4)	2.1 (1.2–3.6)	3.5 (2.6–4.7)	—	**2.1 (1.8–2.6)**	**250,000**
Cigars	1.4 (1.0–2.0)	1.6 (1.1–2.2)	1.1 (0.7–1.7)	1.9 (1.1–3.1)	2.4 (1.6–3.4)	—	**1.5 (1.2–2.0)**	**170,000**
Smokeless tobacco	1.2 (0.9–1.7)	2.4 (1.8–3.2)	1.6 (1.0–2.3)	—	3.2 (2.4–4.2)	—	**1.9 (1.5–2.4)**	**210,000**
Hookah	1.1 (0.7–1.5)	1.6 (1.1–2.4)	0.6 (0.3–1.1)	1.8 (1.1–3.1)	2.7 (1.9–3.9)	—	**1.4 (1.0–1.8)**	**150,000**
Pipe tobacco	—	—	—	—	—	—	**0.4 (0.3–0.7)**	**40,000**
Bidis	—	—	—	—	—	—	**0.3 (0.2–0.5)**	**30,000**
Any tobacco product**	4.8 (4.0–5.8)	6.4 (5.4–7.4)	5.1 (4.0–6.4)	4.9 (3.6–6.5)	7.7 (6.3–9.4)	—	**5.6 (5.0–6.4)**	**670,000**
≥2 tobacco products^††^	2.0 (1.6–2.6)	2.7 (2.0–3.7)	1.9 (1.4–2.7)	2.5 (1.6–3.8)	3.7 (2.7–5.0)	—	**2.4 (2.0–2.9)**	**280,000**
Any combustible tobacco product^§§^	3.2 (2.5–4.0)	3.5 (2.7–4.4)	2.4 (1.8–3.1)	3.9 (2.7–5.7)	5.3 (4.2–6.6)	—	**3.4 (2.8–4.0)**	**390,000**

**Abbreviation:** CI = confidence interval; E-cigarettes = electronic cigarettes.

* Past 30-day use of e-cigarettes was determined by asking, “During the past 30 days, on how many days did you use e-cigarettes?” Past 30-day use of cigarettes was determined by asking, “During the past 30 days, on how many days did you smoke cigarettes?” Past 30-day use of cigars was determined by asking, “During the past 30 days, on how many days did you smoke cigars, cigarillos, or little cigars?” Past 30-day use of hookah was determined by asking, “During the past 30 days, on how many days did you smoke tobacco in a hookah or waterpipe?” Smokeless tobacco was defined as use of chewing tobacco, snuff, dip, snus, and/or dissolvable tobacco products. Past 30-day use of smokeless tobacco was determined by asking the following question for use of chewing tobacco, snuff, and dip: “During the past 30-days, on how many days did you use chewing tobacco, snuff, or dip?,” and the following question for use of snus and dissolvable tobacco products: “In the past 30 days, which of the following products did you use on at least one day?” Responses from these questions were combined to derive overall smokeless tobacco use. Past 30-day use of pipe tobacco (not hookah) and bidis were determined by asking, “In the past 30 days, which of the following products have you used on at least one day?”

^†^ Blacks, whites, and others are non-Hispanic; Hispanic persons could be of any race.

^§^ Estimated total number of users was rounded down to the nearest 10,000 persons.

^¶^ Data are statistically unreliable because samples size was <50 or relative standard error was >0.3.

** Any tobacco product use was defined as use of any tobacco product (e-cigarettes, cigarettes, cigars, smokeless tobacco, hookah, pipe tobacco, and/or bidis) on at least one day in the past 30 days.

^††^ ≥2 tobacco products use was defined as use of two or more tobacco products (e-cigarettes, cigarettes, cigars, smokeless tobacco, hookah, pipe tobacco, and/or bidis) on at least one day in the past 30 days.

^§§^ Any combustible tobacco product use was defined as use of cigarettes, cigars, hookah, pipe tobacco, and/or bidis on at least one day in the past 30 days.

Among middle school students, 5.6% (0.67 million) currently used any tobacco product, including 2.4% (0.28 million; 41.8% of current tobacco product users) who currently used ≥2 tobacco products, and 3.4% (0.39 million; 58.2% of current tobacco product users) who currently used any combustible tobacco product (Table). The most commonly used tobacco product among middle school students was e-cigarettes (3.3%), followed by cigarettes (2.1%), smokeless tobacco (1.9%), cigars (1.5%), hookah (1.4%), pipe tobacco (0.4%), and bidis (0.3%). Any tobacco product use was 6.4% among males and 4.8% among females. E-cigarettes were the most commonly used product among Hispanic (4.0%), white (3.4%), and black (2.2%) middle school students.

Among high school students, a nonlinear decrease occurred in the current use of any tobacco product from 2011 (24.2%) to 2017 (19.6%). Nonlinear decreases also occurred in the current use of ≥2 tobacco products (12.0% to 9.2%) and any combustible tobacco product (21.8% to 12.9%). By product, linear decreases occurred for cigarettes (15.8% to 7.6%), cigars (11.6% to 7.7%), and smokeless tobacco (7.9% to 5.5%); nonlinear decreases occurred for pipe tobacco (4.0% to 0.8%) and bidis (2.0% to 0.7%) (Figure 1). E-cigarette use among high school students increased nonlinearly during 2011–2017 (1.5% to 11.7%).

**Figure 1:**
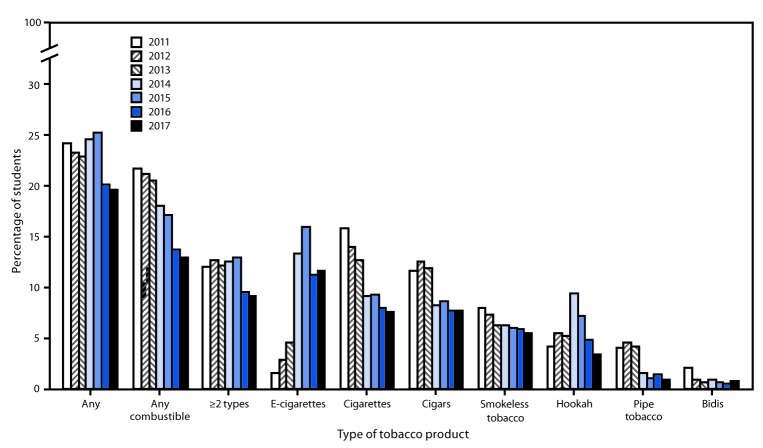
Estimated percentage of high school students who currently use any tobacco product,* any combustible tobacco product,^†^ ≥2 tobacco products,^§^ and selected tobacco products — National Youth Tobacco Survey, United States, 2011–2017^¶^,**,^††^ * Use of any tobacco product was defined as use of electronic cigarettes (e-cigarettes), cigarettes, cigars, smokeless tobacco, hookah, pipe tobacco, and/or bidis on at least one day in the past 30 days. ^†^ Use of any combustible tobacco product was defined as use of cigarettes, cigars, hookah, pipe tobacco, and/or bidis on at least one day in the past 30 days. ^§^ Use of ≥2 tobacco products was defined as use of two or more of the following tobacco products: e-cigarettes, cigarettes, cigars, smokeless tobacco, hookah, pipe tobacco, and/or bidis on at least one day in the past 30 days. ^¶^ During 2016–2017, current use of hookah and pipe tobacco decreased significantly (p<0.05). ** During 2011–2017, current use of cigarettes, cigars, and smokeless tobacco exhibited linear decreases (p<0.05). Current use of any tobacco product, any combustible tobacco product, ≥2 types of tobacco products, pipe tobacco, and bidis exhibited nonlinear decreases (p<0.05). Current use of e-cigarettes exhibited a nonlinear increase (p<0.05). Current use of hookah exhibited a nonlinear change (p<0.05). ^††^ Beginning in 2015, the definition of smokeless tobacco included chewing tobacco/snuff/dip, snus, and dissolvable tobacco to better reflect this class of tobacco products. Thus, estimates for individual smokeless tobacco products (chewing tobacco/snuff/dip, snus, and dissolvable tobacco) are not reported. This definition was applied across all years (2011–2017) for comparability purposes.

Among middle school students, linear decreases occurred in current use of any tobacco product (7.5% to 5.6%), ≥2 tobacco products (3.8% to 2.4%), and any combustible tobacco product (6.4% to 3.4%). By product, linear decreases occurred for cigars (3.5% to 1.5%), smokeless tobacco (2.7% to 1.9%), and pipe tobacco (2.2% to 0.4%); nonlinear decreases occurred for cigarettes (4.3% to 2.1%) and bidis (1.7% to 0.3%). Nonlinear increases occurred in use of e-cigarettes (0.6% in 2011 to 3.3% in 2017) and hookah (1.0% to 1.4%) among middle school students (Figure 2).

**Figure 2:**
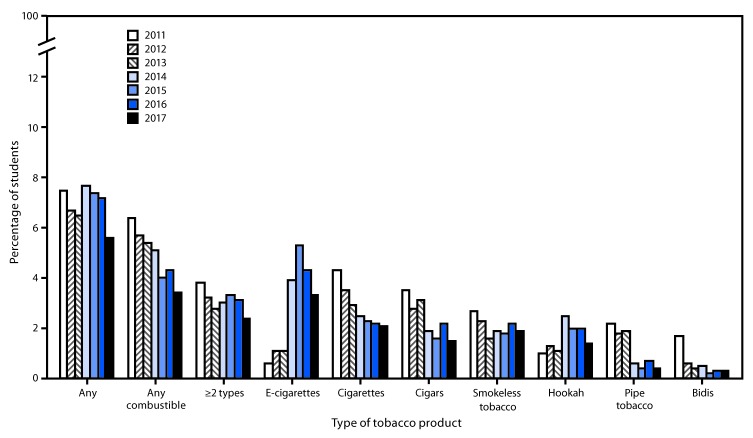
Estimated percentage of middle school students who currently use any tobacco product,* any combustible tobacco product,^†^ ≥2 tobacco products,^§^ and selected tobacco products — National Youth Tobacco Survey, United States, 2011–2017^¶^,**,^††^ * Use of any tobacco product was defined as use of electronic cigarettes (e-cigarettes), cigarettes, cigars, smokeless tobacco, hookah, pipe tobacco, and/or bidis on at least one day in the past 30 days. ^†^ Use of any combustible tobacco product was defined as use of cigarettes, cigars, hookah, pipe tobacco, and/or bidis on at least one day in the past 30 days. ^§^ Use of ≥2 tobacco products was defined as use of two or more of the following tobacco products: e-cigarettes, cigarettes, cigars, smokeless tobacco, hookah, pipe tobacco, and/or bidis on at least one day in the past 30 days. ^¶^ During 2016–2017, current use of any tobacco product, e-cigarettes, and hookah decreased significantly (p<0.05). ** During 2011–2017, current use of any tobacco product, any combustible tobacco product, ≥2 tobacco products, cigars, smokeless tobacco, and pipe tobacco exhibited significant linear decreases (p<0.05). Cigarettes and bidis exhibited significant nonlinear decreases (p<0.05). E-cigarettes and hookah exhibited significant nonlinear increases (p<0.05). ^††^ Beginning in 2015, the definition of smokeless tobacco included chewing tobacco/snuff/dip, snus, and dissolvable tobacco to better reflect this class of tobacco products. Thus, estimates for individual smokeless tobacco products (chewing tobacco/snuff/dip, snus, and dissolvable tobacco) are not reported. This definition was applied across all years (2011–2017) for comparability purposes.

During 2016–2017, among high school students, decreases occurred in current use of hookah (4.8% to 3.3%) and pipe tobacco (1.4% to 0.8%). Among middle school students, decreases occurred in current use of any tobacco product (7.2% to 5.6%), e-cigarettes (4.3% to 3.3%), and hookah (2.0% to 1.4%).

## Discussion

Among U.S. middle and high school students, the current use of any tobacco product decreased during 2011–2017. However, in 2017, approximately one in five high school students (2.95 million) and one in 18 middle school students (0.67 million) currently used a tobacco product. Since 2014, e-cigarettes have been the most commonly used tobacco product among both middle and high school students. Furthermore, approximately one in two high school students who used a tobacco product and two in five middle school students who used a tobacco product reported using ≥2 tobacco products. Among youths, symptoms of nicotine dependence are increased in multiple tobacco product–users compared with those in single product–users (*3*).

Tobacco prevention and control strategies at the national, state, and local levels might have contributed to the reduction in any tobacco product use in recent years, including tobacco product price increases, comprehensive smoke-free policies, media campaigns warning about the risks for youth tobacco product use, and youth access restrictions (*1*,*2*,*4*). However, several factors continue to promote and influence tobacco product use among youths, including exposure to tobacco product advertising and imagery through various media, as well as the availability of flavored tobacco products (*2*,*5*,*6*). Sustained and targeted interventions to address these factors could help prevent and reduce all forms of tobacco use among U.S. youths (*1*,*2*,*4*). In March 2018, the Food and Drug Administration issued an advance notice of proposed rulemaking to obtain information related to the role that flavors play in tobacco product use (*7*).

The findings in this report are subject to at least four limitations. First, findings might not be generalizable to all youths; those who are home-schooled, have dropped out of school, or are in detention centers are not included in this survey. Second, data were self-reported and might be subject to recall and response bias. Third, changes in the wording and placement of survey questions for certain tobacco products during 2011–2017 might limit comparability of responses between years. Finally, data on some tobacco products were unavailable for certain years (e.g., roll-your-own cigarettes), which might result in underestimation of overall tobacco product use.

The sustained implementation of population-based strategies, in coordination with the regulation of tobacco products by FDA (*8*), are critical to reducing all forms of tobacco product use and initiation among U.S. youths (*1*,*2*,*4*). Strategies to reduce youth tobacco product use include increasing the price of tobacco products, implementing comprehensive smoke-free policies, implementing advertising and promotion restrictions and national public education media campaigns, and raising the minimum age of purchase for tobacco products to 21 years (*1*,*4*,*9*).

**Summary**

**What is already known about this topic?**

Tobacco use is the leading cause of preventable disease and death in the United States; nearly all tobacco use begins during youth and young adulthood.

**What is added by this report?**

During 2011–2017, prevalence of current use of any tobacco product decreased from 24.2% to 19.6% among high school students and from 7.5% to 5.6% among middle school students. Electronic cigarettes were the most commonly used tobacco product among high school (11.7%) and middle school students (3.3%) in 2017.

**What are the implications for public health practice?**

Sustained implementation of population-based strategies, in coordination with Food and Drug Administration regulation of tobacco products, are critical to reducing tobacco product use and initiation among U.S. youths.

## Footnotes

* https://www.cdc.gov/tobacco/data_statistics/surveys/nyts/index.htm.

† Beginning in 2015, the definition of smokeless tobacco included chewing tobacco/snuff/dip, snus, and dissolvable tobacco to better reflect this class of tobacco products. Thus, estimates for individual smokeless tobacco products (chewing tobacco/snuff/dip, snus, and dissolvable tobacco) are not reported.

§ During 2011–2013, e-cigarette use was assessed by the question “In the past 30 days, which of the following products have you used on at least one day?” and the response option, “Electronic cigarettes or e-cigarettes such as Ruyan or NJOY.” In 2014, current use of e-cigarettes was assessed by the question “During the past 30 days, on how many days did you use e-cigarettes such as Blu, 21st Century Smoke, or NJOY?” During 2015–2017, e-cigarette questions were preceded by an introductory paragraph defining the product. In 2015, current use of e-cigarettes was assessed by the question “During the past 30 days, on how many days did you use electronic cigarettes or e-cigarettes?” In 2016 and 2017, current use of e-cigarettes was assessed by the question “During the past 30 days, on how many days did you use e-cigarettes?”

¶ During 2011–2015, current hookah smoking was assessed by the question “In the past 30 days, which of the following products have you used on at least one day?” Hookah was the fourth or fifth response option during 2011–2013, the first option in 2014, and the fourth option in 2015. During 2016–2017, hookah questions were preceded by an introductory paragraph defining the product; current hookah smoking was assessed by the question “In the past 30 days, on how many days did you smoke tobacco in a hookah or waterpipe?”

** During 2011–2013, pipe tobacco use was assessed by the question “During the past 30 days, on how many days did you smoke tobacco in a pipe?” During 2014–2017, current use of pipe tobacco was assessed by the question “In the past 30 days, which of the following products have you used on at least one day?” and the response option “Pipes filled with tobacco (not waterpipe).” Pipe tobacco was the second response option available in 2014, the fifth option in 2015, and the second option during 2016–2017.

†† A test for linear trend was significant if an overall statistically significant decrease or increase occurred during the study period. Data also were assessed for the presence of quadratic trends. A significant quadratic trend indicated that the rate of change accelerated or decelerated across the study period.
